# Grapevine Aquaporins: Gating of a Tonoplast Intrinsic Protein (TIP2;1) by Cytosolic pH

**DOI:** 10.1371/journal.pone.0033219

**Published:** 2012-03-12

**Authors:** Luís Leitão, Catarina Prista, Teresa F. Moura, Maria C. Loureiro-Dias, Graça Soveral

**Affiliations:** 1 CBAA, Instituto Superior de Agronomia, Universidade Técnica de Lisboa, Lisbon, Portugal; 2 UMR Bioemco, équipe IBIOS, Université Paris Est Créteil, Créteil, France; 3 REQUIMTE, Dep. Química, FCT-UNL, Caparica, Portugal; 4 Dep. Bioquímica e Biologia Humana, Faculdade de Farmácia, Universidade de Lisboa, Lisbon, Portugal; New Mexico State University, United States of America

## Abstract

Grapevine (*Vitis vinifera* L.) is one of the oldest and most important perennial crops being considered as a fruit ligneous tree model system in which the water status appears crucial for high fruit and wine quality, controlling productivity and alcohol level. *V. vinifera* genome contains 28 genes coding for aquaporins, which acting in a concerted and regulated manner appear relevant for plant withstanding extremely unfavorable drought conditions essential for the quality of berries and wine. Several *Vv* aquaporins have been reported to be expressed in roots, shoots, berries and leaves with clear cultivar differences in their expression level, making their *in vivo* biochemical characterization a difficult task. In this work *V. vinifera cv.* Touriga nacional *Vv*Tn*PIP1;1*, *Vv*Tn*PIP2;2* and *Vv*Tn*TIP2;1* were expressed in yeast and water transport activity was characterized in intact cells of the transformants. The three aquaporins were localized in the yeast plasma membrane but only *Vv*TnTIP2;1 expression enhanced the water permeability with a concomitant decrease of the activation energy of water transport. Acidification of yeast cytosol resulted in loss of *Vv*TnTIP2;1 activity. Sequence analysis revealed the presence of a His^131^ residue, unusual in TIPs. By site directed mutagenesis, replacement of this residue by aspartic acid or alanine resulted in loss of pH_in_ dependence while replacement by lysine resulted in total loss of activity. In addition to characterization of *Vv*Tn aquaporins, these results shed light on the gating of a specific tonoplast aquaporin by cytosolic pH.

## Introduction

Plant growth and development are dependent on the tight regulation of water uptake and transport across cellular membranes and tissues at whole plant level. Depending on the environmental conditions and water balance, plants can modify the relative contribution of apoplastic and cell-to-cell water-flow pathways across the tissues to adjust the overall hydraulic conductivity [1]. Aquaporins are essential in the cell-to-cell pathway as their presence allows not only higher water permeability but also control and regulation of water flow [1], providing a fine tuning of the hydraulic conductivity of this pathway in response to biotic and abiotic stresses. With 28 members in *Vitis vinifera* [2], plants appear to express a much higher and diverse number of aquaporin homologues than mammalian or yeast. These proteins belong to the family of Major Intrinsic Proteins (MIPs). Based on their sequence similarity and on their main subcellular location plant aquaporins were organized in seven subfamilies: the plasma membrane intrinsic proteins (PIPs), the tonoplast intrinsic proteins (TIPs), the nodulin26-like intrinsic proteins (NIPs), the small and basic intrinsic proteins (SIPs), the GlpF-like intrinsic proteins (GIPs), the hybrid intrinsic proteins (HIPs) and the uncategorized X intrinsic proteins (XIPs) [3]. However, individual aquaporins cannot easily be assigned to homogeneous subcellular compartments, being reported that each organelle may be equipped with combinations of different isoforms and that, although predominant in one cellular localization, the same subfamily can be expressed differently according to plant tissue. This diversity suggests a putative role of these channels in different cell types or tissues for survival and development upon a wide range of conditions, but their specific physiological relevance still remains poorly understood [4]. Furthermore, aquaporin activity can be regulated by gating factors or mechanisms [5] such as osmotic solutes [6], pressure pulses [7], membrane tension [8], [9], cytosolic pH and pCa^2+^ [10], [11] and post translational mechanisms like phosphorylation [12]–[15], glycosylation [16], methylation and acetylation [17] and heterotetramer formation [18]. Several stress factors were reported to affect plant aquaporin activity such as oxidative stress [19], NaCl [20], dehydration and excessive watering and chilling [21].

Grapevine (*Vitis vinifera* L.) is one of the oldest and most important perennial crops being considered as a fruit ligneous tree model system [22] in which the water status appears crucial for high fruit and wine quality, controlling productivity and alcohol level [23]. Furthermore, flooding of soils results in oxygen deprivation (anoxia) of plant roots during raining season, or after irrigation. One early response of plants to anoxia and other environmental stresses is the inhibition of root water permeability leading to a down-regulation of water uptake [14]. It was also reported that flooding induced anoxia led to reduced water uptake in roots. It was proposed that intracellular acidification, arising as a consequence of anoxia, would mediate inhibition of water channels [24]. Direct evidence for pH regulation was observed in *Arabidopsis* PIPs heterologously expressed in *Xenopus laevis* oocytes [14], [25]. Moreover, it was shown that replacement of a histidine residue by an alanine or aspartic acid in loop D at position 197 prevented pH sensitivity [14], [24]. *V. vinifera* PIPs (Chardonnay and Grenache cultivars) have shown water transport activity when individually expressed in *Xenopus* [21]. Several *Vv*PIPs and *Vv*TIPs have been reported to be expressed in roots, shoots, berries and leaves [2], [26], [27] with clear cultivar differences in their expression level [21], making their *in vivo* biochemical characterization a difficult task.

Due to its intrinsic low water plasma membrane permeability, yeast has been reported as a suitable system for heterologous individual aquaporin expression [28]. Moreover, the activity of heterologous water channels anchored in the membrane of a *aqy-null* strain can be powerfully characterized by stopped flow spectroscopy. Yeasts can be maintained in a diversity of environments and even the composition of the interior of the cell can be controlled, creating a variety of conditions to study the specificity and regulation of putative water channels. The use of a fluorescence self-quenching methodology for assessing water transport in intact cells (without removing the cell wall) [29] represents an increased value for the study of yeast expressed plant aquaporins. This methodology opens new perspectives to measure water permeability in minimally disturbed cells that are quite stable during a rather long experiment [29].

In the present work, we describe the molecular cloning process of two *Vv*PIPs (*Vv*TnPIP1;1 and *Vv*TnPIP2;2) and one *Vv*TIP (*Vv*TnTIP2;1) from *V. vinifera cv.* Touriga nacional and their characterization using the *Saccharomyces cerevisiae* heterologous expression system. The cultivar Touriga nacional was chosen as representative of Portuguese traditional cultivars with an important recent commercial value and deserving much interest from enologists throughout the world. Moreover, the cellular localization of each aquaporin was identified in yeast and the osmotic stress tolerance of these strains was evaluated. Finally, functional characterization (water permeability and activation energy of water transport) of *Vv*TnTIP2;1 was performed, focusing on a putative aquaporin intracellular pH regulation mechanism.

## Results

### Cloning and molecular characterization of *V. vinifera* PIP1;1, PIP2;2 and TIP2;1

Oligonucleotide primers designed from 5′UTR and 3′UTR of *V. vinifera cv.* Cabernet sauvignon aquaporin *PIP1;1*, *PIP2;2* and *TIP2;1* sequences, available on the Grape Genome database from Genoscope, were used for amplification of aquaporin cDNAs from mRNAs extracted from *V. vinifera cv.* Touriga nacional *callus* cells. This procedure led to the isolation of four full length cDNAs encoding the corresponding putative aquaporins. A multiple alignment of the deduced amino acid sequences is shown in Figure S1. All the deduced sequences present the characteristic topology of six highly hydrophobic transmembrane spanning helices connected by five loops with the N- and C-terminal sequences on the cytosolic side of the membrane. The highly conserved SGxHxNPA sequence in the first half of the protein and the second highly conserved region in the second half of the protein, forming part of the MIP family signature, are also present [30].

The genes corresponding to the cDNAs cloned in this study were clearly identified as encoding two proteins belonging to the PIP subfamily and two belonging to the TIP subfamily. Each of the PIP aquaporins showed an obvious and specific resemblance with PIP1 and PIP2 *V. vinifera* sequences subfamily (Figure S1). Accordingly, the phylogenetic analysis of these sequences clearly showed the consistent distribution of *V. vinifera cv*. Touriga nacional aquaporins, clustered within the group of *V. vinifera* PIP1, PIP2 and TIP2 aquaporins (Figure S2). The nomenclature proposed for the grapevine aquaporins was established according to the results of multiple alignments with MIP genes from *V. vinifera cv. Cabernet sauvignon*, *Pinot noir* and *Syrah* (Figure S2).

The comparative analysis of *Vv*TnPIP1;1, *Vv*TnPIP2;2 and *Vv*TnTIP2;1 amino acid sequences from Touriga nacional cultivar with other similar proteins from other *V. vinifera* cultivars was also performed. The aquaporin sequences were mostly identical to all the similar proteins, excepting that a V276M substitution was found in *Vv*TnPIP1;1 and that a M235I substitution was found in *Vv*TnTIP2;1, both localized at the C-terminal region (Figure S3).

Search for signatures characteristic of atypical substrates transport, as proposed by Hove *et al.* [31], was also performed. Interestingly, *Vv*TnTIP2;1 sequence revealed all the characteristic residues indicating that besides water it may also permeate ammonia. As for *Vv*TnPIP1;1 and *Vv*TnPIP2;2, both present all the key residues of MIPs for H_2_O_2_ transport.

To characterize the three putative *V. vinifera* aquaporins, plasmids harboring the corresponding ORFs, driven by the MET25 promoter and tagged with the GFP sequence at their 3′ end were constructed (Table 1). The plasmids were retrieved, their sequences confirmed by restriction analysis and sequencing and were used to transform *S. cerevisiae aqy-null* strain. For negative control, the same strain was transformed with the empty plasmid pUG35. Transformants containing *Vv*Tn*PIP1;1* (LL0.P11), *Vv*Tn*PIP2;2* (LL0.P22), and *Vv*Tn*TIP2;1* (LL0.T21) were obtained. An additional mutated *Vv*Tn*TIP2;1* in which glutamic acid E146 was replaced by a glycine residue was also cloned (LL0.T21.E146G). Representative colonies from each clone were used for heterologous expression studies.

**Table 1:** Plasmids and strains used in this study.

Plasmids		
Name in this work	Relevant characteristics	Source
empty plasmid	pUG35	U. Güldener and J. H. Hegemann
P11	pUG35-*Vv*Tn*PIP1;1*	This study
P22	pUG35-*Vv*Tn*PIP2;2*	This study
T21	pUG35-*Vv*Tn*TIP2;1*	This study
T21.E146G	pUG35-*Vv*Tn*TIP2;1* E146G	This study
T21.H131A	pUG35-*Vv*Tn*TIP2;1* H131A	This study
T21.H131D	pUG35-*Vv*Tn*TIP2;1* H131D	This study
T21.H131K	pUG35-*Vv*Tn*TIP2;1* H131K	This study

Yeast strains		
Name in this work	Genotype	Source
*S. cerevisae YSH1172*	10560-6B *MATa leu2::hisG trp1::hisG his3::hisG ura3-52 aqy1::KanMX4 aqy2::HIS3*	P. Van Dijck
LL0	YSH1172+pUG35	This study
LL0.P11	YSH1172+pUG35-*Vv*Tn*PIP1;1*	This study
LL0.P22	YSH1172+pUG35-*Vv*Tn*PIP2;2*	This study
LL0.T21	YSH1172+pUG35-*Vv*Tn*TIP2;1*	This study
LL0.T21.E146G	YSH1172+pUG35-*Vv*Tn*TIP2;1* E146G	This study
LL0.T21.H131A	YSH1172+pUG35-*Vv*Tn*TIP2;1* H131A	This study
LL0.T21.H131D	YSH1172+pUG35-*Vv*Tn*TIP2;1* H131D	This study
LL0.T21.H131K	YSH1172+pUG35-*Vv*Tn*TIP2;1* H131K	This study

**Figure 1:**
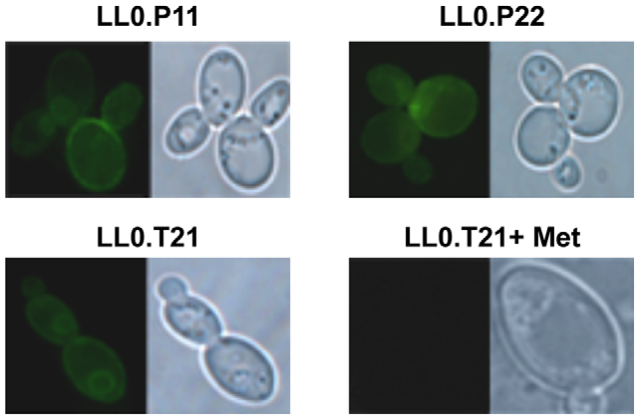
Localization of GFP-tagged *V. vinifera* aquaporins in yeast plasma membrane. Epifluorescence (left panels) and phase contrast (right panels) images of *S. cerevisiae aqy-null* strains YSH1172 transformed with centromeric plasmids harboring genes from *V. vinifera cv*. Touriga nacional aquaporins. Cells, grown in YNB medium, show aquaporin localization at the yeast plasma membrane: (A) *Vv*TnPIP1;1 (LL0.P11), (B) *Vv*TnPIP2;2 (LL0.P22) and (C) *Vv*TnTIP2;1 (LL0.T21). In (D), expression of *Vv*TnTIP2;1 was repressed by methionine supplementation in the growth medium.

GFP-tagging confirmed the plasma membrane localization of *Vv*TnPIP1;1, *Vv*TnPIP2;2, *Vv*TnTIP2;1 proteins (Figure 1), and the presence of similar amounts of the tagged proteins. Membrane localization and expression level was also confirmed for the mutated version of *Vv*TnTIP2;1 harbored by LL0.T21.E146G (data not shown).

The regulation of expression levels by MET25 promoter was tested. The strains were grown in YNB supplemented with methionine and the fluorescence resulting from the presence of the aquaporin-GFP tagged proteins was drastically reduced, confirming the repression of gene expression under the control of MET25 promoter (Figure 1).

### *V. vinifera* PIP and TIP expression affects yeast growth under osmotic stress

**Figure 2:**
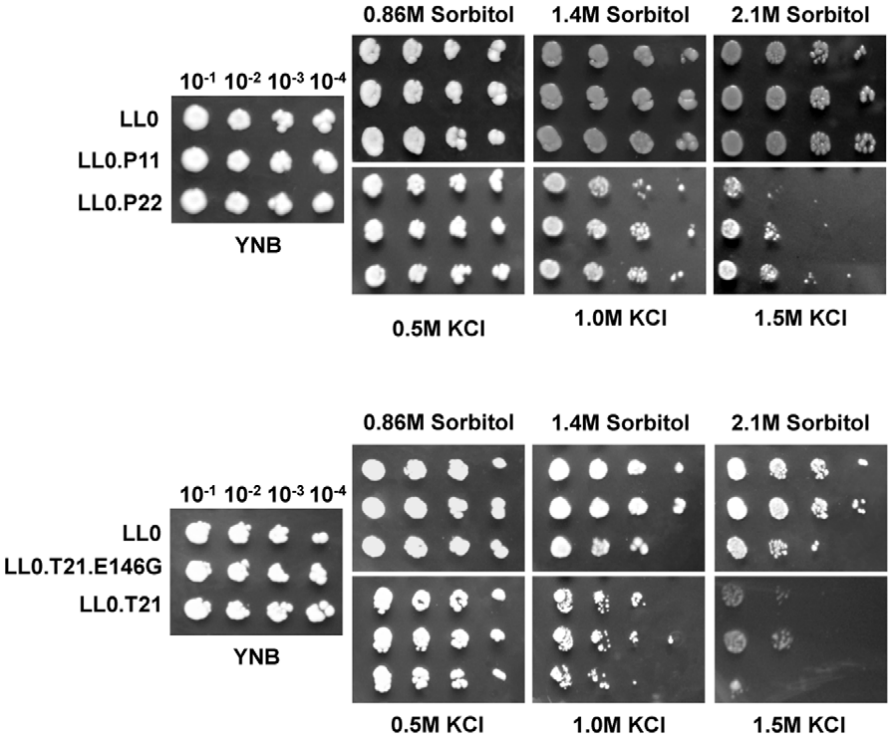
Effect of *V. vinifera* aquaporins expression on yeast growth under osmotic stress. Strains expressing (A) *Vv*TnPIPs (LL0.P11 and LL0.P22) and (B) *Vv*TnTIPs (LL0.T21 and LL0.T21.E146G) were grown in YNB medium, serially diluted in sterile water and spotted onto YNB plates containing the indicated osmo-equivalent concentrations of sorbitol or KCl. Images were taken after 1 week incubation at 28°C. PIPs enhanced the ability to grow under osmotic stress conditions while the native TIP had the opposite effect. Identical results were obtained in three independent experiments. (see text for details).

The expression of the aquaporin genes inserted into pUG35 plasmid was not toxic to yeast, since the growth of all strains was similar to the strain transformed with the empty plasmid (LL0) both in YNB solid (Figure 2) and liquid medium (data not shown).

Although the relationship between water permeation and osmotic stress is still not clear, previous reports show that the absence of water channels in yeast improved resistance to osmotic stress [32]. These results led us to examine the effect of plant aquaporin expression on yeast osmotic stress tolerance. The osmotic stress tolerance of strains expressing *Vv*TnPIP1;1, *Vv*TnPIP2;2 and *Vv*TnTIP2;1 was examined. Figure 2 shows growth tests performed in solid YNB medium (pH 5.5) containing osmo-equivalent concentrations of sorbitol or KCl (0.86 M sorbitol or 0.5 M KCl, 1.4 M sorbitol or 1 M KCl and 2.1 M sorbitol or 1.5 M KCl). Strain LL0 was used as a negative control. Comparing the strains containing the different cloned aquaporins, the transformants bearing PIPs behaved in a similar way. A clear enhancement of the ability to grow under osmotic stress conditions was observed when these strains were grown in medium above 0.5 M KCl. On the other hand, strain LL0.T21 expressing *Vv*TnTIP2;1 presented the opposite phenotype, showing a clear growth inhibition in the presence of concentrations above 0.5 M KCl, as compared to negative control LL0 (Figure 2B). The behavior of LL0.T21.E146G, harboring the mutated version of *Vv*TnTIP2;1 was similar to that of LL0. In general, the parallel effect of sorbitol was less evident.

### Functional assessment of water transport

**Figure 3:**
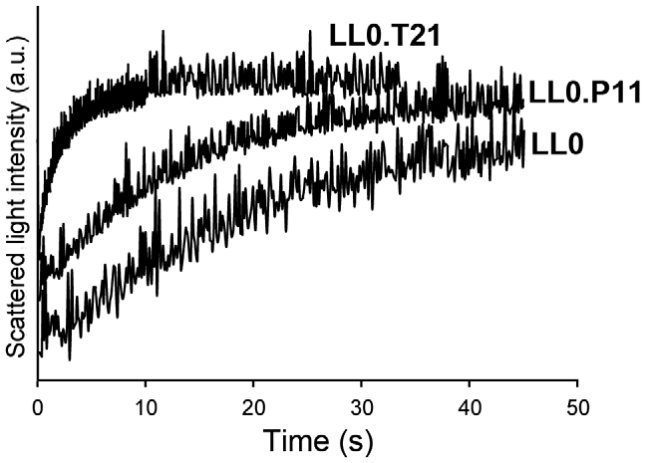
Water permeability assessment of *V. vinifera* aquaporins expressed in yeast. Stopped-flow fluorescence signals obtained for yeast strains expressing *Vv*TnPIP1;1 (LL0.P11) and *Vv*TnTIP2;1 (LL0.T21) are compared with the negative control (LL0). Cells were loaded with CFDA (1 mM in isosmotic solution) at pH 5 and subjected to a hyperosmotic shock with sorbitol (tonicity = 1.25). A faster cell volume change can be observed for *Vv*TnTIP2;1. The traces correspond to an average of 4 to 6 individual time courses of fluorescence intensity at 9°C.

To analyze aquaporin activity, yeast strains expressing *V. vinifera* water channels were loaded with CFDA and challenged with a hypertonic sorbitol solution in a stopped flow device. The change in the fluorescence signal due to water efflux was used to calculate the osmotic permeability coefficient (*P_f_*) and the activation energy (*E_a_*) of water transport. As shown in Figure 3, the expression of *Vv*TnTIP2;1 (LL0.T21) led to a large increase of the shrinking rate as compared to the non-expressing strain (LL0). However, expression of *Vv*TnPIP1;1 (LL0.P11) and *Vv*TnPIP2;2 (LL0.P22, not shown) did not affect the time course of water efflux.

**Figure 4:**
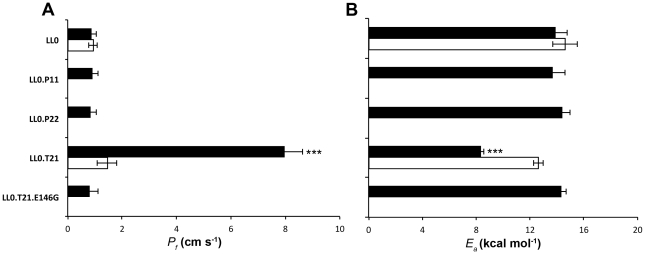
Water transport activity in yeast strains expressing *Vv*TnPIPs and *Vv*TnTIPs. (A) Osmotic permeability coefficient (*P_f_*) and (B) activation energy (*E_a_*) of water transport were measured at pH 5. An 8 fold increase in water permeability is observed for *Vv*TnTIP2;1 expression (LL0.T21) (black bar), which was repressed by methionine incubation (white bars). No increase of water permeability was detected by expression of *Vv*TnPIP1;1 (LL0.P11), *Vv*TnPIP2;2 (LL0.P22) or a mutated *Vv*TnTIP2;1 (LL0.T21.E146G), as compared to the negative control (LL0). The *E_a_* values determined are in good agreement with the *P_f_* measured. Data are mean ± SEM of at least three independent experiments. ***P<0.001.

Figure 4 compares the *P_f_* values obtained at 9°C and the *E_a_* for the different strains. The enhance in water permeability conferred by *Vv*TnTIP2;1 expression is clearly depicted (8.0±0.7×10^−4^ cm s^−1^), while no activity for *Vv*TnPIP1;1 (1.0±0.2×10^−4^ cm s^−1^) nor for *Vv*TnPIP2;2 (0.9±0.2×10^−4^ cm s^−1^) expressing strains could be detected when compared to the LL0 strain (0.9±0.2×10^−4^ cm s^−1^). The increase in permeability is consistent with the decrease in *E_a_* for the LL0.T21 strain (14.0±0.9 kcal mol^−1^ (58.2±3.7 kJ mol^−1^) for LL0 and 8.3±0.2 kcal mol^−1^ (34.7±0.8 kJ mol^−1^) for LL0.T21), therefore assuring that the pathway for water flow is being affected due to an increase in aquaporin activity. When methionine was added to the growth medium repressing the gene expression, the activity of *Vv*TnTIP2;1 was diminished in the LL0.T21 strain (*P_f_* of 1.5±0.4×10^−4^ cm s^−1^) with a concomitant increase in the *E_a_* (12.7±0.4 kcal mol^−1^ (53.1±1.7 kJ mol^−1^)). Methionine addition had no effect in LL0 strain permeability. Interestingly, the water transport activity obtained with the strain expressing the mutated *Vv*TnTIP2;1 (TIP21.E146G) was not different from LL0 (*P_f_* of 0.8±0.1×10^−4^ cm s^−1^ and *E_a_* of 14.3±0.4 kcal mol^−1^ (59.8±1.7 kJ mol^−1^)), suggesting that the replacement of glutamic acid residue at position 146 by a glycine caused loss of function of the tonoplast aquaporin.

### Cytosolic pH regulates *V. vinifera* TIP activity

Previous reports indicate that some mammalian aquaporins are regulated by external pH [33] and that plants respond to conditions that lead to a decrease in cytosolic pH with reduction of water membrane permeability [34]. Also, the direct evidence for pH regulation of *A. thaliana* and *V. vinifera* PIP aquaporins heterologously expressed in *Xenopus* oocytes [14], [25] led us to study pH regulation of *V. vinifera cv.* Touriga nacional water channels when cloned in yeast.

The intracellular pH of yeast transformants was determined as indicated in Methods. An external acidification from pH 6.8 to 5.0 induced an intracellular pH (pH_in_) decrease from 6.8 to 6.1. When cells were incubated with 4 mM benzoic acid at pH 5.0, a further marked drop in pH_in_ from 6.1 to 4.8 was observed.

**Figure 5:**
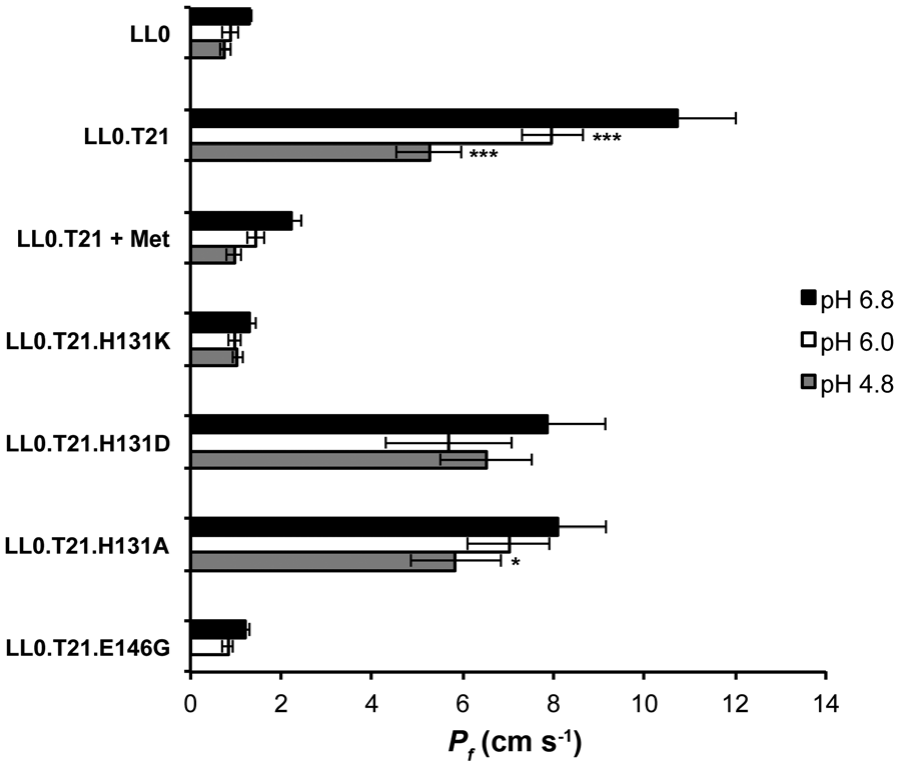
Water permeability of yeast strains expressing mutated *Vv*TnTIP2;1. Osmotic permeability coefficient (*P_f_*) was measured at intracellular pH 6.8 (full bars), pH 6.1 (white bars) and pH 4.8 (gray bars). Yeast cells expressing the wild type *Vv*TnTIP2;1 (in the absence and in the presence of methionine) and the mutated *Vv*TnTIP2;1 forms (H/K, H/D, H/A) are compared. Strain LL0.T21.E146G is also shown. The yeast strain LL0 stands for a negative control (see text for details). Data are mean ± SEM of at least three independent experiments. *P<0.05, **P<0.01, ***P<0.001.

Acidification of the yeast cytosol reduced water permeability in the LL0.T21 strain as shown in Figure 5. The 10-fold higher *P_f_* found for LL0.T21 (10.7±1.3×10^−4^ cm s^−1^) compared to LL0 (1.3±0.04×10^−4^ cm s^−1^), decreased when pH_in_ dropped from 6.8 to 6.1 (7.9±0.6×10^−4^ cm s^−1^). At pH_in_ 4.8, a further decrease in activity was detected (5.2±0.7×10^−4^ cm s^−1^). This observed ca. 50% reduction of permeability was consistent with the ca. 50% increase in the *E_a_* values for the same experimental acidic conditions as shown in Table 2. The improved aquaporin activity of LL0.T21 strain was repressed by methionine incubation, reaching values similar to the negative controls at all pH_in_ tested. Strains LL0 and the mutated LL0.T21E146G containing a non-active TIP2;1 aquaporin found to be pH insensitive (*P_f_* of 1.2±0.1×10^−4^ cm s^−1^) were used as negative controls.

**Table 2:** Activation energy (*E_a_*) of water transport at different intracellular pH.

	*E_a_* [kcal mol^−1^ (kJ mol^−1^)]		
Strain	pH_in_ 6.8	pH_in_ 6.1	pH_in_ 4.8
LL0	14.4±0.7 (60.3±2.9)	13.9±0.8 (58.2±3.3)	13.3±0.8 (55.7±3.3)
LL0.T21	6.9±0.4 (28.9±1.7)	8.4±0.2 (35.2±0.8)	10.7±0.6 (44.8±2.5)
LL0.T21 + Met	12.0±0.3 (50.2±1.3)	12.6±0.4 (57.8±1.7)	15.0±0.4 (62.9±1.7)
LL0.T21.H131K	13.5±0.3 (56.6±1.3)	14.5±0.4 (60.8±1.7)	16.2±0.3 (67.9±1.3)
LL0.T21.H131D	8.0±0.1 (33.5±0.4)	8.8±0.4 (36.8±1.7)	8.2±0.3 (34.4±1.3)
LL0.T21.H131A	7.5±0.1 (31.4±0.4)	8.0±0.3 (33.5±1.3)	9.4±0.3 (39.3±1.3)
LL0.T21.E146G	14.4±0.3 (60.3±1.3)	14.3±0.4 (59.9±1.7)	nd

nd: not determined; Values are mean ± SEM of at least three independent experiments.

*E_a_* of water transport was evaluated from the slope of Arrhenius plots (ln*P_f_* as a function of 1/T) in yeast strains expressing *V. vinifera* wild type and mutated *Vv*TnTIP2;1.

Yeast strains expressing *Vv*TnPIP1;1 and *Vv*TnPIP2;2 were tested at extracellular pH 5 and pH 6.8. As expected, both strains showed low *P_f_* (1.3±0.2×10^−4^ cm s^−1^) not dependent on extracellular pH, and consistently high *E_a_* values (14.1±1.3 kcal mol^−1^ (60.0±5.4 kJ mol^−1^)).

From these data we conclude that pH_in_ rather than pH_out_ regulates aquaporin activity, since the addition of 4 mM benzoic acid at pH_out_ 5.0, changing pH_in_ without changing pH_out_, resulted in a decreased permeability. At pH 6.8, benzoic acid (8 mM) had no effect on pH_in_ and no effect on permeability (not shown) supporting the idea that pH_in_ and not benzoic acid itself affected aquaporin activity.

### Role of histidine 131 residue in TIP activity

*Vv*TnTIP2 sequences possess a histidine residue (His^131^) in loop D, common in PIPs (His^193^ in spinach, His^196^ in tobacco, His^199^ in strawberry plant and His^194^ in *A. thaliana* aquaporin PIP2;1) but not in TIPs from other plants. Such histidine residues were reported to have a key function in pH-dependent aquaporin gating [14], [15], [24]. The presence of the His^131^ residue at loop D of *Vv*TnTIP2;1 together with a leucine in the following vicinity, as described for plant PIPs [35], led us to look for a possible role of this histidine in the pH-dependent regulation of *Vv*TnTIP2;1 aquaporin activity. We introduced point mutations to replace His^131^ by Asp (H131D), Ala (H131A), and Lys (H131K), and after confirming the membrane localization of the mutated *Vv*TnTIP2;1 proteins, water transport activity was evaluated under conditions where yeast pH_in_ was adjusted as indicated above.

Fast water uptake was observed when the variant H131D was expressed; however, aquaporin activity was not pH dependent (*P_f_* of (7.8±1.3), (5.7±1.4) and (6.5±1.0)×10^−4^ cm s^−1^ for pH_in_ 6.8, 6.1 and 4.8, respectively) (Figure 5). The variant H131A showed a poor pH dependent activity compared to the wild-type protein (*P_f_* of (8.1±1.1), (7.0±0.9) and (5.8±1.0)×10^−4^ cm s^−1^ for pH_in_ 6.8, 6.1 and 4.8, respectively). The variant H131K lost the water transport ability through the mutated aquaporin for every pH tested (*P_f_* of (1.3±0.1), (1.0±0.1) and (1.0±0.2)×10^−4^ cm s^−1^ for pH_in_ 6.8, 6.1 and 4.8, respectively). These results were corroborated by a consistent pattern of E_a_ values (Table 2).

## Discussion

*Vitis vinifera*, the grapevine originated in the eastern Mediterranean area, is generally cultivated in soils where water is the main limiting factor in agriculture, under conditions of drought and salinity stress. Although the adaptation of this plant to dry climates is well known, the molecular bases of water transport in grapevine still need further elucidation. Many genes encoding aquaporins were isolated from different plants including ligno-cellulosic species [22]. The recent release of *Vitis vinifera* genome allowed the identification of 28 putative aquaporin genes among which 8 coding for PIPs and 10 coding for TIPs have been found [2]. However, only the expression of a single or/and combination of each aquaporin isoforms in heterologous systems unequivocally allows the identification of its individual properties [36].

In this study we have cloned and individually characterized two *Vv*TnPIPs and one *Vv*TnTIP by heterologous expression in an *aqy-null* yeast strain. Their sequences show all the typical characteristics of the corresponding sub-family for water permeation and additionally some other key residues that suggest permeation of other putative substrates [31]. Therefore, their specificity for atypical substrates should be further investigated.

The osmotic water permeabilities were assessed in intact yeast cells expressing these aquaporins by stopped-flow fluorescence using a volume sensitive dye. This technique enables the assessment of water transport in yeast cells with intact physiological functions of the cell wall, facilitating the biochemical characterization of aquaporins from foreign systems, such as plants, by heterologous expression. Taking advantage of this walled cell permeability assessment and in order to individually characterize aquaporins, the *E_a_* was calculated enabling to distinguish between the increase in aquaporin activity and the increase in lipid bilayer permeability [37]. It is well known that changes in pH affect the lipid bilayer permeability [24], [38] possibly affecting accuracy of aquaporin activity measurements. Therefore, when comparing permeabilities measured at distinct pHs, a second criterion such as the *E_a_* should be taken into account. In this study, the *E_a_* values determined were in good agreement with the *P_f_* measured, indicating that the aquaporin activity rather than the bilayer fluidity is being evaluated.

All the cloned aquaporins were successfully expressed in a *S. cerevisiae aqy-null* mutant and their localization at the plasma membrane was confirmed by GFP fluorescence. Interestingly, the growth phenotypes of the yeast strains transformed with each of the two PIPs and one TIP were different and in accordance with previous studies showing that the absence of active aquaporins improve yeast osmotic tolerance [32]. Under osmotic stress conditions, the strains harboring the PIPs exhibited an improved growth, whereas the strain expressing *Vv*Tn*TIP2;1* exhibited growth inhibition. Although the right localization of *Vv*TnPIP1;1 and *Vv*TnPIP2;2 at the yeast plasma membrane was confirmed, no increase in water conductance was observed. An incorrect insertion or even a defect in the oligomerization state of these aquaporins into the yeast plasma membrane may explain the lack of activity observed. However, since an improved growth phenotype under osmotic stress was depicted for strains expressing either *Vv*TnPIP, one may speculate that these proteins might be involved in the transport of atypical substrates as suggested by the performed sequence analysis. Also, the possibility that PIPs co-expression is required for function, as previously reported for maize [39] and for *Beta vulgaris* [36], cannot be discarded.

The permeability induced by *Vv*TIP2;1 expression was roughly ten-fold higher compared to the aquaporin null strain, consistent with a lower *E_a_* and dependent on cytosolic acidification. A similar pH effect was reported using molecular dynamic simulations and point mutations on a conserved His residue in the intracellular loop D of PIP aquaporins from tobacco [24] and *A. thaliana* [14]. The protonation of this specific His residue leading to the pore closure was correlated with a consequent loss of water transport through these proteins. The analysis of amino acid sequence of *Vv*TnTIP2;1 revealed an equivalent His residue localized in loop D and facing the cytosol (His^131^), suggesting that TIP2;1 might sense and be regulated by cytosolic pH; this idea was confirmed by the absence of pH regulation in *At*TIP1;1 [14] and in *Vv*TIP1;1 [25] which lack the correspondent His. To examine the molecular basis of *Vv*TnTIP2;1 sensitivity to pH, we investigated the effects of point mutations in the H^131^ residue that might be involved in this regulation. This aromatic basic and polar residue was individually mutated to an acidic and polar residue (Asp, H131D), a neutral and non-polar residue (Ala, H131A) and an aliphatic basic and polar residue (Lys, H131K). A marked decrease in water permeability was observed for the H131K mutant; in spite of belonging to the same amino acid class, the Lys residue shows a longer hydrocarbon chain with a higher pKa (10.53 for Lys *vs.* 6.10 for His), implying that not only a steric effect but also its permanent protonation at cytosolic pH may be responsible for the loss of water permeability. The H131D mutant showed a similar water channel activity for the same cytosolic pH, but with loss of pH sensitivity, a result that can be expected from its acidic lateral chain with a pKa of 3.86, preventing its protonation. As for the H131A mutant, this non-polar small amino acid was not expected to contribute to channel blockage; indeed, only a minor effect on pH sensitivity that cannot be assigned to the residue charge or structure was observed.

Evidence for aquaporin gating by cytosolic pH has been reported for *B. vulgaris* roots using an enriched fraction of tonoplast membrane vesicles [40]. In this case when pH dropped from 8.3 to 5.6, permeability decreased 42%. A similar inhibition was observed for *Vv*TnTIP2;1 in the present study when cytosolic pH dropped from 6.8 to 4.8, the activity being specifically dependent on cytosolic pH. Conversely, *At*TIP5;1 activity was shown to be dependent on extracellular pH [41]. On the contrary, when expressed in oocytes, *Vv*TIP1;1 from Cabernet Sauvignon was shown to be insensitive to pH [25]. Apparently, different patterns of pH regulation can be found among tonoplast aquaporins.

In conclusion, these results suggest that His^131^ in the D loop segment of *Vv*TnTIP2;1 is involved in aquaporin gating, being the first clear evidence of a pH regulation for a TIP2;1. These data may provide a clue for a coordinated regulation of TIPs in plant tissues under anoxic pressure, as previously suggested [14], [42]. Further studies will be required to clarify the role of *V. vinifera* aquaporins aiming to untangle the mechanisms involved in ligno-cellulosic plant water balance.

## Materials and Methods

### Plant Material

*Vitis vinifera* cv. Touriga nacional (kindly provided by S. Amâncio and S. Tavares, ISA-TUL) was used. Cell suspensions were obtained by adapting *V. vinifera callus* to liquid culture as described in [43]. Briefly, 4 g *callus* tissue was dispersed in 50 ml of liquid MS [44] (Dufemie, Haarlem, NL) supplemented with 2.5 µM 2,4-D, 1 µM kinetin, 5 g/l PVP-40T, 20 g l^−1^ sucrose, pH 5.7, in 250 ml flasks. The cultures growing in the dark, at 25°C, in a rotary shaker at 100 rpm were sub-cultured weekly by diluting 25 ml culture into 25 ml of fresh medium.

### Yeast strains, maintenance and growth conditions

The yeast strains and plasmids used in this work are listed in Table 1. All *Saccharomyces cerevisiae* strains are derivatives of *S. cerevisiae* 10560-6B *MAT*α *leu2::hisG trp1::hisG his3::hisG ura3-52 aqy1::KanMX4 aqy2::HIS3*; (further indicated as *aqy-null*). *S. cerevisiae aqy-null* was used as recipient strain in complementation experiments with the plasmids listed in Table 1. *Escherichia coli* DH5α [45] was used for routine propagation of the plasmids. *E. coli* DH5α strain was routinely maintained in Luria-Bertani medium (LB) at 37°C; ampicilin (100 µg ml^−1^) and 5-bromo-4-chloro-3-indolyl-ß-D-galactopyranoside (X-Gal, 4 µg ml^−1^) were used as supplements [46] when required. The recipient yeast strain was maintained in YPD medium (5 g l^−1^ yeast extract, 10 g l^−1^ peptone, 20 g l^−1^ glucose and 20 g l^−1^ agar). Transformant strains were maintained and grown in YNB medium without amino acids (DIFCO) with 2% (w/v) glucose (and 2% (w/v) agar for solid medium) supplemented with the adequate requirements for prototrophic growth [47]. When gene expression was to be reduced to its minimum level, an additional supplementation with 6 mM methionine was used.

The ability of yeast strains to grow under osmotic stress was assessed on solid YNB medium supplied with sorbitol or KCl (pH 5) to the desired final concentrations. Cells were grown in liquid YNB medium with orbital shaking, at 28°C up to OD_640_≈1, corresponding to 1×10^7^ cells/ml. Multi-well plates were prepared with serial 10-fold dilutions of the original culture and plates were inoculated with 3 µl drops using a replica platter for 96-well plates device and incubated at 28°C. Growth was recorded after 1 and 2 weeks.

For stopped-flow assays, the same medium was used. Cells were grown with orbital shaking, at 28°C up to OD_640_≈1, harvested by centrifugation (10 000×g; 3 min; 25°C), re-suspended in YPD medium (6 g l^−1^ wet weight) and incubated 1 hour at 28°C. Cells were then harvested by centrifugation (10 000×g; 3 min; 4°C), washed, and re-suspended in ice cold 1.4 M sorbitol (0.3 g ml^−1^ wet weight).

### Heterologous expression of *V. vinifera* aquaporins in *S. cerevisiae*

*RNA extraction and cDNA preparation* - Total RNA was extracted from *V. vinifera callus* cells using RNeasy plant Mini Kit (Qiagen, Hilden, Germany) according to the manufacturer protocol. All RNA samples were treated with RNase free DNase I (Qiagen, Hilden, Germany) and quantified using absorption of U.V. light at 260 nm. cDNAs were synthesized from mRNAs using Superscript III first strand Synthesis system for RT-PCR, priming with oligo-d(T)12-18 following manufacturer's instructions (Invitrogen), and were used for PCR reaction using the primers described in Table S1. Full-length CDS were obtained using specific primers designed within the 5′ and the 3′ non-coding region of each cDNAs. PCR products were sequenced by automatic sequencing ABI Prism DNA sequencer (Perkin-Elmer), analyzed using Blast tools and compared with other plant known aquaporin sequences focusing specially on *V. vinifera* available sequences.

*Cloning aquaporin genes* - Forward and reverse aquaporin primers modified to incorporate restriction sites for *Xba*I (underlined) and *Cla*I (underlined), respectively, were used to amplify DNA fragments containing full-length ORFs encoding the respective aquaporin (Table S1). PCR amplification was carried out in an Eppendorff thermocycler with DNA polymerase from Finnzymes (annealing temperature according to the primer characteristics). The amplified products were sequenced, digested with *Cla*I and *Xba*I, purified using the purification kit “GFX PCR DNA and Gel Band Purification” (GE Healthcare) and cloned into the corresponding restriction sites of pUG35 digested by the same restriction enzymes, behind MET25 promoter and in frame with GFP sequence and CYC1-T terminator. Cloning was performed according to standard protocols described in [46]. Constructs were named according to Table 1. In addition, a mutated *Vv*Tn*TIP2;1* in which glutamic acid E146 was replaced by a glycine residue G146 (TIP21.E146G) was also obtained. The plasmids were cloned into a DH5α *E. coli* strain, propagated, subjected to extraction and restriction analysis and sequenced.

Transformation of *S. cerevisiae aqy-null* strain was performed by the lithium acetate method described in [48]. Transformants were selected on YNB medium without uracil. *E. coli* plasmid isolation was performed by alkaline extraction as described [49] and modified [46]. For plasmid isolation from yeasts, the procedure described by [50] was used. Agarose gel electrophoresis and restriction site mapping were performed according to standard methods [46].

*Sequence analyses* - DNA and protein sequences for comparative analysis were obtained from the Grape Genome Browser (http://www.genoscope.cns.fr/externe/GenomeBrowser/Vitis/) [51] and Genebank [52], [53]. Multiple protein sequence alignments were generated using the ClustalX [54] and Bioedit [55] programs and phylogenetic trees were obtained by using the Phylogeny.fr program [56].

*Cellular localization of V. vinifera aquaporins* - Exponentially grown cells were spotted onto microscope slides and observed with an Olympus AX70 fluorescent microscope. For GFP visualizing, a U-MWB fluorescent cube was used with excitation filter 450–480 nm and barrier filter 515 nm.

*Site directed mutagenesis* - For site directed mutagenesis, plasmid extracted from LL0.T21 was used as template for mutagenic PCR using primers described in Table S1. Each primer contained the proper nucleotide mutation and a 45 bp tail in order to promote homologous recombination. An internal fragment of TIP2;1 aquaporin cDNA was removed by digestion with *Xba*I and *Bst*I restriction enzymes. The PCR mutagenized product and digested plasmid were purified as described above. *S. cerevisiae* was transformed simultaneously with both DNA fragments, allowed to recombine and selected in medium without uracil. Yeast colony PCR products were sequenced.

### Cell volume assay

Equilibrium cell volumes were obtained by loading cells with CFDA under a fluorescent microscope equipped with a digital camera as previously described [29]. Cells were assumed to have a spherical shape with a diameter calculated as the average of the maximum and minimum dimensions of each cell. Sorbitol osmotic shocks of increasing tonicity were imposed on a microscope slide and an average of 6 pictures with 4–6 cells each were taken before (V_o_) and within 10 to 40 s after the osmotic challenge (V_∞_). The tonicity of the osmotic shock is defined as the ratio of the final to initial osmolarity of the outside medium, Λ = (osm_out_)_∞_/(osm_out_)_o_.

### Measurement of osmotic permeability coefficient

The stopped-flow technique was used to monitor cell volume changes induced by osmotic shocks, in cells loaded with a concentration-dependent self-quenching fluorophore [29]. Cells were pre-loaded for 10 min at 30°C with the nonfluorescent precursor 5-and-6-carboxyfluorescein diacetate (CFDA, 1 mM in isosmotic solution) that is cleaved by intracellular nonspecific esterases generating the fluorescent form, expected to remain mainly in the cytoplasm. Although some of the probe may be either accumulated in the vacuole or exported to the medium [57], this effect can be neglected in face of the very rapid water flow [29]. As the cells shrink in response to osmotic changes, the concentration of the entrapped fluorophore increases with a change in the fluorescence output [29]. To avoid pH interference in fluorescence during the osmotic shock, cell suspensions and osmotic solutions were buffered with K^+^-citrate/KH_2_PO_4_ 50 mM at the selected pH.

Experiments were performed on a HI-TECH Scientific PQ/SF-53 stopped-flow apparatus, which has a 2 ms dead time, controlled temperature, interfaced with an IBM PC/AT compatible 80386 microcomputer. Four runs were usually stored and analyzed in each experimental condition. In each run, 0.1 ml of cell suspension (initial osmolarity (osm_out_)_o_ = 1.4 M) was mixed with an equal amount of hyperosmotic sorbitol solution (final tonicity = 1.5) to reach an inwardly directed solute gradient and induce an outward water flow responsible for cell volume change. Fluorescence was excited using a 470 nm interference filter and detected using a 530 nm cut-off filter. The time course of volume change was followed by fluorescence quenching CFDA. The recorded fluorescence signals were fitted to a single exponential from which the rate constant (*k*) was calculated. The osmotic water permeability coefficient, *P_f_*, was estimated from the linear relationship between *P_f_* and *k* [58], *P_f_* = *k*(*V_o_*/*A*)(1/*V_w_*(*osm_out_*)*_∞_*), where *V_w_* is the molar volume of water, *V_o_*/*A* is the initial volume to area ratio of the cell population, and (*osm_out_*)*_∞_* is the final medium osmolarity after the osmotic shock.

### Activation energy of water transport

Stopped-flow experiments were performed at temperatures ranging from 9 to 37°C. The activation energy (*E_a_*) of water transport was evaluated from the slope of Arrhenius plots (ln*P_f_* as a function of 1/T).

### pH effect on aquaporin activity

In order to evaluate pH effect on aquaporin activity, adjustments of intracellular pH (pH_in_) of yeast cells expressing *V. vinifera* aquaporins were undertaken by varying extracellular pH (pH_out_) and by adding benzoic acid, which promotes intracellular acidification due to the passive diffusion of the non-dissociated form of the acid followed by dissociation inside the cell [59]. Cells were washed and incubated under three distinct conditions: pH_out_ 6.8, pH_out_ 5, and pH_out_ 5 plus 4 mM benzoic acid in ice cold 1.4 M sorbitol and re-suspended in the same buffer with the same final biomass concentration in each suspension. Cells were kept on ice under these conditions for 90 minutes before stopped flow assays. pH_in_ was calculated from the relative distribution of labeled ^14^[C]propionic acid [60] using the same conditions described above.

### Statistical analysis

In all experiments, at least three independent batches of cultures were grown and analyzed. Cell volume, permeabilities and pH effect were repeated in at least three independent experiments. The data were analyzed using either Student's t test or ANOVA and are presented as mean values ± standard error of the means (SEM). P<0.05 was considered to be statistically significant.

## Supporting Information

Figure S1 **Alignment of the deduced amino acid sequences encoded by the cDNAs isolated in this study with (A) PIP and (B) TIP aquaporin sequences from** ***Vitis vinifera cultivar*** **Cabernet sauvignon available in Genoscope database.** Deduced amino acid sequences were compared using the ClustalX multiple alignment program [54]. Identical amino acid residues are shaded in gray and the boxed areas refer to MIP family signature sequences. Accession numbers are VvCsPip1;1-GSVIVP00029248001, VvCsPIP1;2-GSVIVP00026881001, VvCsPIP1;3-GSVIVP00000433001, VvCsPIP1;4-gb|ABH09325.1, VvCsPIP1;5-GSVIVP00026882001, VvCsPIP2;2-GSVIVP00036133001, VvCsPIP2;1-CAN75442, VvCsPIP2;3-GSVIVP00023192001, VvCsTIP1;1-GSVIVP00018548001, VvCsTIP1;2-GSVIVP00000605001, VvCsTIP1;3-GSVIVP00022146001, VvCsTIP1;4-GSVIVP00024394001, VvCsTIP2;1-GSVIVP00034350001, VvCsTIP2;2-GSVIVP00012703001, VvCsTIP3;1-GSVIVP00013854001, VvCsTIP4;1-GSVIVP00032441001, VvCsTIP5;1-GSVIVP00029946001 and VvCsTIP5;2-GSVIVP00019170001 (for Cabernet sauvignon cultivar sequences); and VvTnPIP1;1-HQ913643, VvTn2;2-HQ913642 and VvTnTIP2;1-HQ913640 (for Touriga nacional cultivar sequences).
(TIF)

Figure S2 **Dendrogram based on primary protein sequence homology depicting the phylogenetic the relationship between** ***Vv*** **TnPIP1;1,** ***Vv*** **TnPIP2;2 and** ***Vv*** **TnTIP2;1 transporters from** ***Vitis vinifera var.*** Touriga nacional and the *Vitis vinifera* aquaporin sequences from Cabernet Sauvignon cultivar available on Grape Genome database from Genoscope. Sequences identified in the present study are framed. For the construction of the phylogenetic tree, multiple amino acid sequence alignments were generated using the ClustalX [54] and Bioedit [55] were used. The resulting tree was drawn by using the Phylogeny.fr program [56]. The proposed nomenclature for the grapevine aquaporins has been established according to the results of multiple alignments with MIP genes from *V. vinifera cv.* Cabernet Sauvignon, Pinot Noir and Syrah (Fig. S3) and in full respect of the current nomenclature. Represented proteins (and corresponding accession numbers) are: VvCsPip1;1-GSVIVP00029248001, VvCsPIP1;2-GSVIVP00026881001, VvCsPIP1;3-GSVIVP00000433001, VvCsPIP1;4-gb|ABH09325.1, VvCsPIP1;5-GSVIVP00026882001, VvCsPIP2;2-GSVIVP00036133001, VvCsPIP2;1- CAN75442, VvCsPIP2;3-GSVIVP00023192001, VvCsTIP1;1- GSVIVP00018548001, VvCsTIP1;2-GSVIVP00000605001, VvCsTIP1;3-GSVIVP00022146001, VvCsTIP1;4-GSVIVP00024394001, VvCsTIP2;1-GSVIVP00034350001, VvCsTIP2;2-GSVIVP00012703001, VvCsTIP3;1-GSVIVP00013854001, VvCsTIP4;1-GSVIVP00032441001, VvCsTIP5;1-GSVIVP00029946001, VvCsTIP5;2-GSVIVP00019170001, VvCsSIP1;1-GSVIVP00025504001, VvCsSIP2;1-GSVIVP00023346001, VvCsNIP1;1-GSVIVP00035815001, VvCsNIP3;1-GSVIVP00022377001, VvCsNIP4;1-GSVIVP00011149001, VvCsNIP5;1-GSVIVP00000446001, VvCsNIP6;1-GSVIVP00033750001, VvCsNIP7;1-GSVIVP00019910001, VvCsNIP8;1-GSVIVP00007127001, VvCsNIP8;2-GSVIVP00003903001 (for Cabernet sauvignon cultivar sequences); VvTnPIP1;1- HQ913643, VvTn2;2- HQ913642 and VvTnTIP2;1- HQ913640 (for Touriga nacional cultivar sequences). NIP3;1 was excluded from the dendogram due to its high level of divergence in relation to all other *V. vinifera* aquaporin sequences.
(TIF)

Figure S3 **Alignment of the deduced amino acid sequences encoded by the cDNAs isolated in this study with (A) PIP1;1, (B) PIP2;2 and (C) TIP2;1 aquaporin sequences from** ***Vitis vinifera*** **cultivars available in databases.** Deduced amino acid sequences were compared using the ClustalX multiple alignment program [54]. Identical amino acid residues are shaded in the same gray scale. Residues that differ between sequences are boxed. Protein database accession numbers are VvTnPIP1;1-HQ913643, VvTn2;2-HQ913642 and VvTnTIP2;1-HQ913640 (for Touriga nacional cultivar sequences), VvCsPIP1;1-GSVIVP00029248001, VvCsPIP2;2-GSVIVP00036133001 and VvCsTIP2;1-GSVIVP00034350001 (for Cabernet sauvignon cultivar sequences), VvPnPIP1;1-XP_002268084.1, VvPnPIP2;2-XP_002279366.1 and VvPnTIP2;1-XP_002284226.1 (for Pinot noir cultivar sequences), VvSyTIP2;1-CAB95746.2 (for Syrah cultivar sequence). The loop D histidine residue and its nearer leucine residue from Touriga nacional cultivar are boxed.
(TIF)

Table S1 **Primer sequences used in this study.**
(DOCX)
