# Supplementary figures and images for: Grapevine Aquaporins: Gating of a Tonoplast Intrinsic Protein (TIP2;1) by Cytosolic pH
Source: PLoS One. 2012 Mar 12;7(3):e33219. doi: 10.1371/journal.pone.0033219 (PMC3299758; doi:10.1371/journal.pone.0033219)

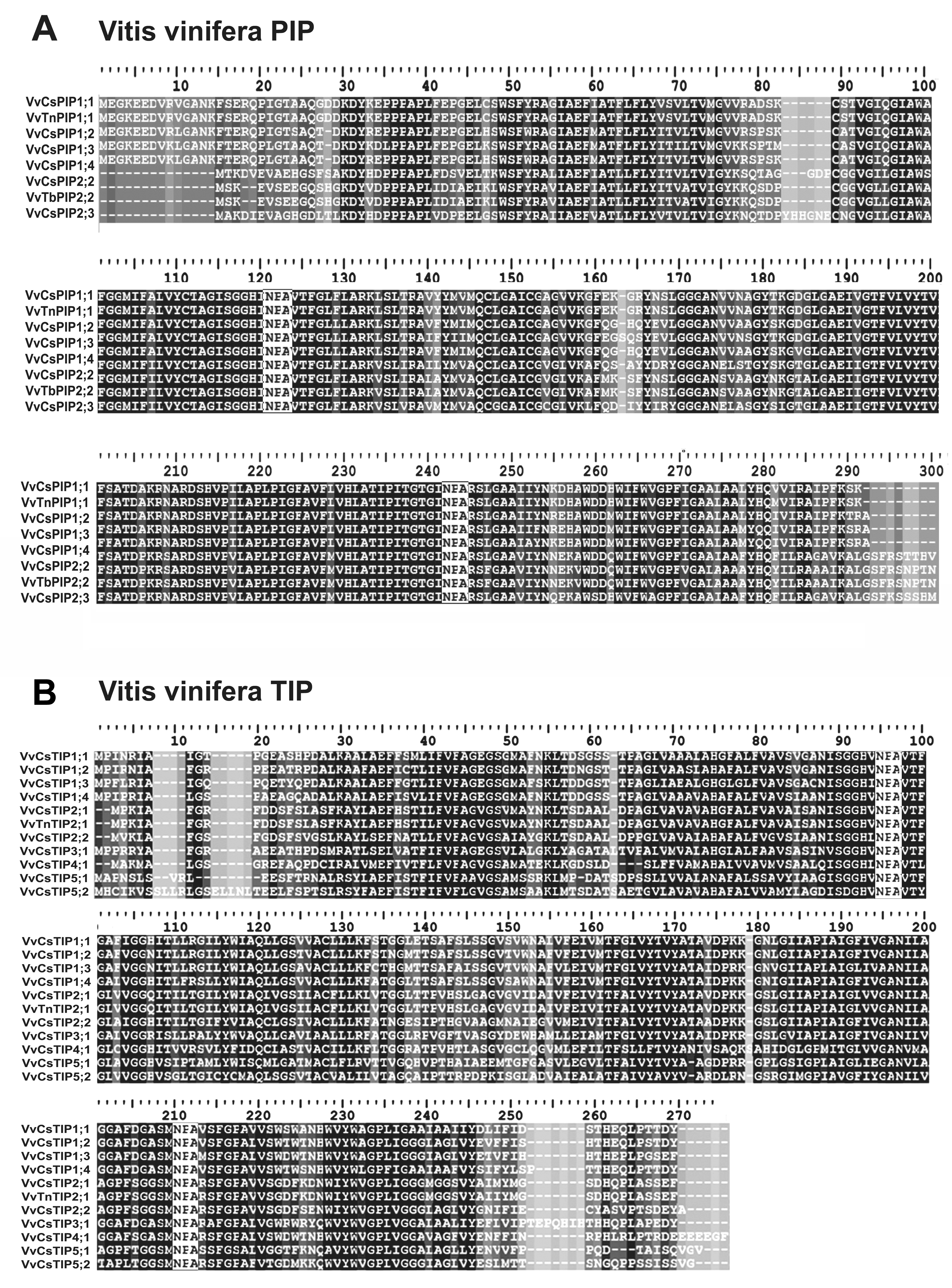

Supplement: Figure S1 — Alignment of the deduced amino acid sequences encoded by the cDNAs isolated in this study with (A) PIP and (B) TIP aquaporin sequences from Vitis vinifera cultivar Cabernet sauvignon available in Genoscope database. Deduced amino acid sequences were compared using the ClustalX multiple alignment program [54]. Identical amino acid residues are shaded in gray and the boxed areas refer to MIP family signature sequences. Accession numbers are VvCsPip1;1-GSVIVP00029248001, VvCsPIP1;2-GSVIVP00026881001, VvCsPIP1;3-GSVIVP00000433001, VvCsPIP1;4-gb|ABH09325.1, VvCsPIP1;5-GSVIVP00026882001, VvCsPIP2;2-GSVIVP00036133001, VvCsPIP2;1-CAN75442, VvCsPIP2;3-GSVIVP00023192001, VvCsTIP1;1-GSVIVP00018548001, VvCsTIP1;2-GSVIVP00000605001, VvCsTIP1;3-GSVIVP00022146001, VvCsTIP1;4-GSVIVP00024394001, VvCsTIP2;1-GSVIVP00034350001, VvCsTIP2;2-GSVIVP00012703001, VvCsTIP3;1-GSVIVP00013854001, VvCsTIP4;1-GSVIVP00032441001, VvCsTIP5;1-GSVIVP00029946001 and VvCsTIP5;2-GSVIVP00019170001 (for Cabernet sauvignon cultivar sequences); and VvTnPIP1;1-HQ913643, VvTn2;2-HQ913642 and VvTnTIP2;1-HQ913640 (for Touriga nacional cultivar sequences). (TIF) [file pone.0033219.s001.tif]

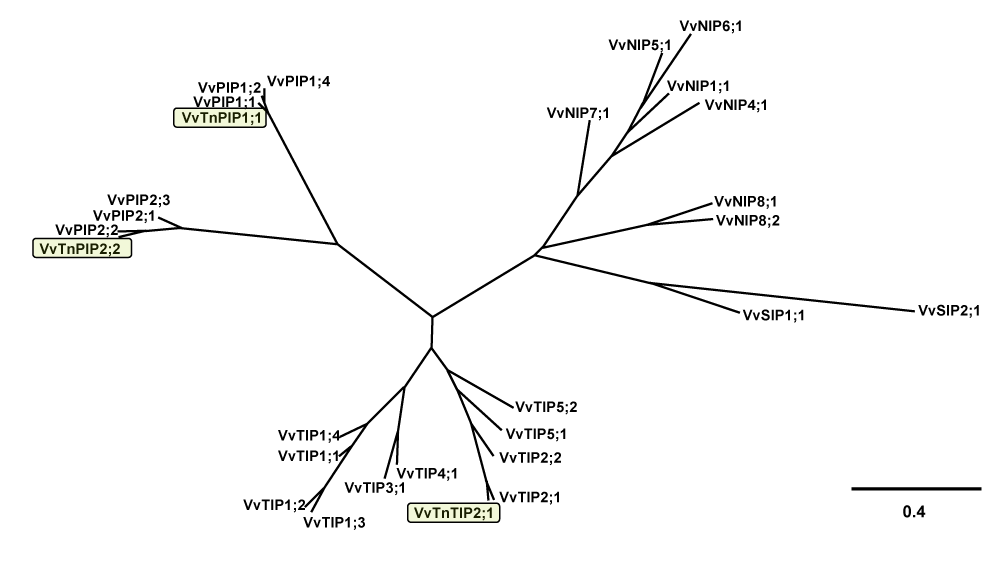

Supplement: Figure S2 — Dendrogram based on primary protein sequence homology depicting the phylogenetic the relationship between Vv TnPIP1;1, Vv TnPIP2;2 and Vv TnTIP2;1 transporters from Vitis vinifera var. Touriga nacional and the Vitis vinifera aquaporin sequences from Cabernet Sauvignon cultivar available on Grape Genome database from Genoscope. Sequences identified in the present study are framed. For the construction of the phylogenetic tree, multiple amino acid sequence alignments were generated using the ClustalX [54] and Bioedit [55] were used. The resulting tree was drawn by using the Phylogeny.fr program [56]. The proposed nomenclature for the grapevine aquaporins has been established according to the results of multiple alignments with MIP genes from V. vinifera cv. Cabernet Sauvignon, Pinot Noir and Syrah (Fig. S3) and in full respect of the current nomenclature. Represented proteins (and corresponding accession numbers) are: VvCsPip1;1-GSVIVP00029248001, VvCsPIP1;2-GSVIVP00026881001, VvCsPIP1;3-GSVIVP00000433001, VvCsPIP1;4-gb|ABH09325.1, VvCsPIP1;5-GSVIVP00026882001, VvCsPIP2;2-GSVIVP00036133001, VvCsPIP2;1- CAN75442, VvCsPIP2;3-GSVIVP00023192001, VvCsTIP1;1- GSVIVP00018548001, VvCsTIP1;2-GSVIVP00000605001, VvCsTIP1;3-GSVIVP00022146001, VvCsTIP1;4-GSVIVP00024394001, VvCsTIP2;1-GSVIVP00034350001, VvCsTIP2;2-GSVIVP00012703001, VvCsTIP3;1-GSVIVP00013854001, VvCsTIP4;1-GSVIVP00032441001, VvCsTIP5;1-GSVIVP00029946001, VvCsTIP5;2-GSVIVP00019170001, VvCsSIP1;1-GSVIVP00025504001, VvCsSIP2;1-GSVIVP00023346001, VvCsNIP1;1-GSVIVP00035815001, VvCsNIP3;1-GSVIVP00022377001, VvCsNIP4;1-GSVIVP00011149001, VvCsNIP5;1-GSVIVP00000446001, VvCsNIP6;1-GSVIVP00033750001, VvCsNIP7;1-GSVIVP00019910001, VvCsNIP8;1-GSVIVP00007127001, VvCsNIP8;2-GSVIVP00003903001 (for Cabernet sauvignon cultivar sequences); VvTnPIP1;1- HQ913643, VvTn2;2- HQ913642 and VvTnTIP2;1- HQ913640 (for Touriga nacional cultivar sequences). NIP3;1 was excluded from the dendogram due to its high level of divergence in relation to [file pone.0033219.s002.tif]

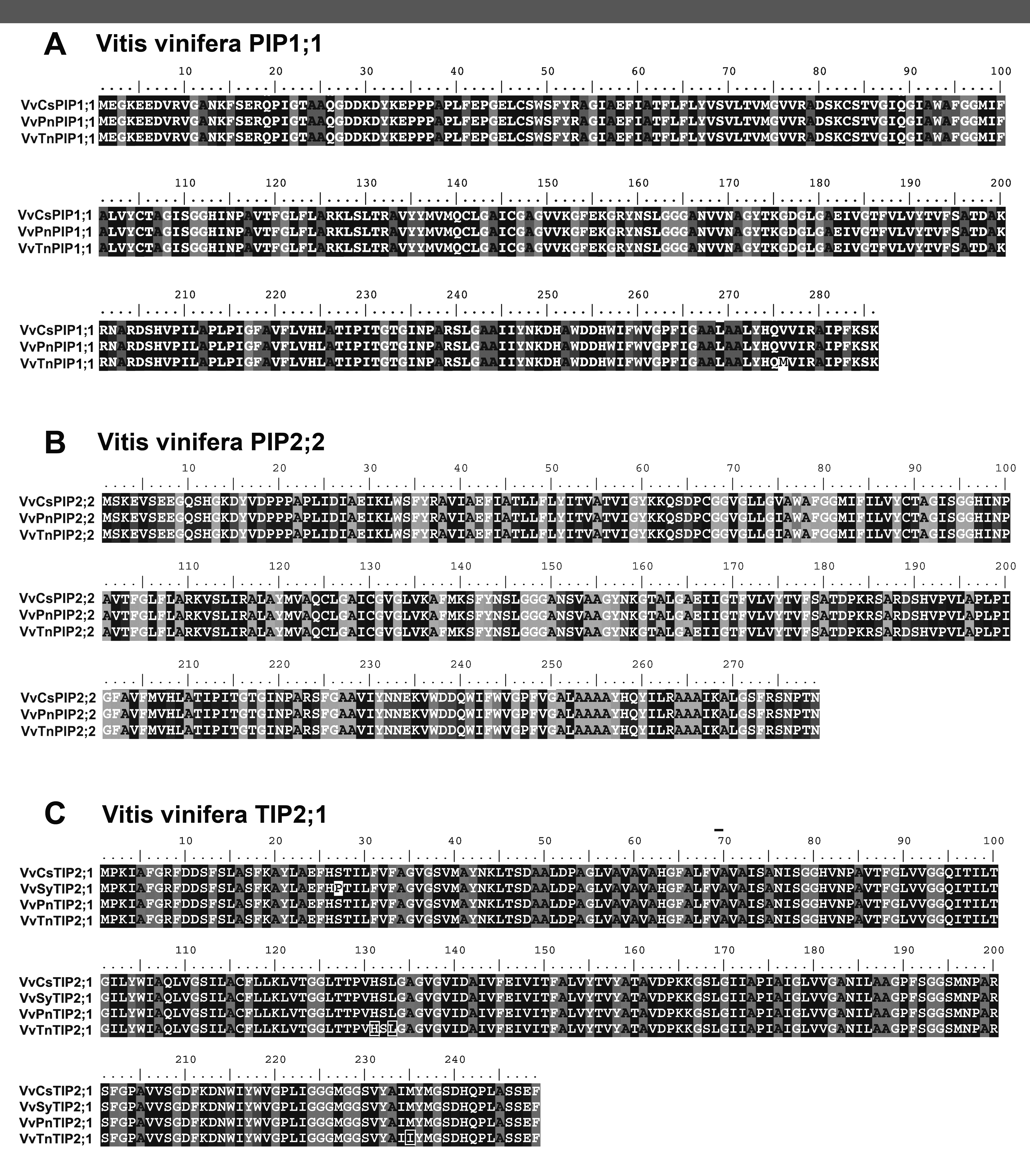

Supplement: Figure S3 — Alignment of the deduced amino acid sequences encoded by the cDNAs isolated in this study with (A) PIP1;1, (B) PIP2;2 and (C) TIP2;1 aquaporin sequences from Vitis vinifera cultivars available in databases. Deduced amino acid sequences were compared using the ClustalX multiple alignment program [54]. Identical amino acid residues are shaded in the same gray scale. Residues that differ between sequences are boxed. Protein database accession numbers are VvTnPIP1;1-HQ913643, VvTn2;2-HQ913642 and VvTnTIP2;1-HQ913640 (for Touriga nacional cultivar sequences), VvCsPIP1;1-GSVIVP00029248001, VvCsPIP2;2-GSVIVP00036133001 and VvCsTIP2;1-GSVIVP00034350001 (for Cabernet sauvignon cultivar sequences), VvPnPIP1;1-XP_002268084.1, VvPnPIP2;2-XP_002279366.1 and VvPnTIP2;1-XP_002284226.1 (for Pinot noir cultivar sequences), VvSyTIP2;1-CAB95746.2 (for Syrah cultivar sequence). The loop D histidine residue and its nearer leucine residue from Touriga nacional cultivar are boxed. (TIF) [file pone.0033219.s003.tif]
