# Supplementary material for: Grapevine Aquaporins: Gating of a Tonoplast Intrinsic Protein (TIP2;1) by Cytosolic pH
Source: PLoS One. 2012 Mar 12;7(3):e33219. doi: 10.1371/journal.pone.0033219 (PMC3299758; doi:10.1371/journal.pone.0033219)
Supplement: Table S1 — Primer sequences used in this study. (DOCX) [file pone.0033219.s004.docx]

**Table S1** – Primer sequences used in this study

| **Primers used for amplification of aquaporin cDNAs** [[2](#_ENREF_2)] | | | | | |  |
| --- | --- | --- | --- | --- | --- | --- |
| **Name** | **cDNAs** | | **Sequence** | | **Description** | |
| P22fwd | *Vv*Tn*PIP2;2* | | CAGAGAGAGAACAGAGAGTGAG | | 5'UTR-forward primer | |
| P22rev | *Vv*Tn*PIP2;2* | | GGAGGGTGTTGTGGGTTT | | 3'UTR-reverse primer | |
| P11fwd | *Vv*Tn*PIP1;1* | | GAGATGGAAGGTAAGGAAGAGGA | | 5'UTR-forward primer | |
| P11rev | *Vv*Tn*PIP1;1* | | AGTGGAATGCTACAGACA | | 3'UTR-reverse primer | |
| T21fwd | *Vv*Tn*TIP2;1* | | TCCTCATCTCTTCATTCATC | | 5'UTR-forward primer | |
| T21rev | *Vv*Tn*TIP2;1* | | CATCACCAACCTCATTCA | | 3'UTR-reverse primer | |
|  |  |  | |  | |  |
| **Primers used for amplification of aquaporin cDNA for cloning step** | | | | | |  |
| **Name** | **cDNAs** | | **Sequence** | | **Description** | |
| P22clfwd | *Vv*Tn*PIP2;2* | | GCTCTAGAGCCAGAGAGAGAACAGAGAGTGAG | | Forward primer | |
| P22clrev | *Vv*Tn*PIP2;2* | | CCATCGATGGTGTTGGTGGGGTTGCTGCGGAA | | Reverse primer | |
| P11clfwd | *Vv*Tn*PIP1;1* | | TGCTCTAGAGCAGAGATGGAAGGTAAGGAAGAGGA | | Forward primer | |
| P11clrev | *Vv*Tn*PIP1;1* | | CCATCGATGGTCTTGGACTTGAAGGGAATGGC | | Reverse primer | |
| T21clfwd | *Vv*Tn*TIP2;1* | | CTAGTCTAGACTAGTCCTCATCTCTTCATTCATC | | Forward primer | |
| T21clrev | *Vv*Tn*TIP2;1* | | CCATCGATGGTGAATTCGCTGGACGCTAGTGG | | Reverse primer | |
|  |  | |  | |  | |
| **Primers used for mutagenesis experiment** | | | | | |  |
| **Name** | **cDNAs** | | **Sequence** | | **Description** | |
| T21.H131Afwd | *Vv*Tn*TIP2;1* H131A, D or K | | GTAATACAGGGTCGTCAGATACATAGATACAATTCTATTACCCCCATCC | | Forward primer | |
| T21.H131Arev | *Vv*Tn*TIP2;1* H131A | | TCCAACCCCAGCTCCAAGACT**TGC**GACGGGAGTCGTCAAGCCTCCT | | Reverse primer | |
| T21.H131Drev | *Vv*Tn*TIP2;1* H131D | | TCCAACCCCAGCTCCAAGACT**GTC**GACGGGAGTCGTCAAGCCTCCT | | Reverse primer | |
| T21.H131Krev | *Vv*Tn*TIP2;1* H131K | | TCCAACCCCAGCTCCAAGACT**CTT**GACGGGAGTCGTCAAGCCTCCT | | Reverse primer | |
